# Asymmetrical vertebral column decancellation for the management of rigid congenital kyphoscoliosis

**DOI:** 10.1186/s12891-020-03558-x

**Published:** 2020-08-17

**Authors:** Fanqi Hu, Wenhao Hu, Xiaoqing Yang, Chunguo Wang, Kai Song, Guoquan Zheng, Xuesong Zhang

**Affiliations:** 1 Medical School of Chinese PLA, Chinese PLA General Hospital, Fuxing Rd. 28, Haidian District, Beijing, China; 2 The Department of Orthopedics, the First Medical Centre, Chinese PLA General Hospital, Fuxing Rd. 28, Haidian District, Beijing, China; 3 Spine Division, Department of Orthopedics, The Fourth Medical Center, Chinese PLA General Hospital, No.51 Fucheng Road, Beijing, China

**Keywords:** Congenital kyphoscoliosis, Vertebral column decancellation, Asymmetrical vertebral column decancellation, Vertebral column resection, Pedicle subtraction osteotomy

## Abstract

**Background:**

Congenital kyphoscoliosis is a disease that often requires surgical treatment. Wedge osteotomies, such as pedicle subtraction osteotomy, are insufficient to correct this complicated rigid deformity. Vertebral column resection yields sufficient correction, but it is an exhaustively lengthy operation with a high risk of major complications. There are few effective and safe techniques for treating rigid congenital kyphoscoliosis. We aimed to investigate the technique of asymmetrical vertebral column decancellation (AVCD) for the treatment of rigid congenital kyphoscoliosis and evaluate the clinical and radiographic results of patients treated with the technique.

**Methods:**

Between January 2013 to June 2017, the data of 31 patients with congenital kyphoscoliosis who underwent single level AVCD were reviewed. Preoperative and postoperative radiographical parameters and the visual analogue scale, Asia Spinal Injury Association, and Scoliosis Research Society-22 scores were documented. The patients were followed up for an average period of 29 months.

**Results:**

The average operative time was 273.9 ± 46.1 min. The average volume of blood loss was 782.3 ± 162.6 ml. The main coronal curve improved from a mean of 81.4° preoperatively to 24.7° at the final follow-up, and the coronal balance improved from 28.9 to 7.6 mm. The degree of local kyphosis improved from a mean of 86.5° to 29.2°, and the sagittal balance improved from 72.3 to 16.9 mm. All clinical outcomes also improved significantly from preoperatively to the final follow-up. No permanent postoperative neurologic complications occurred.

**Conclusion:**

The AVCD surgical procedure corrects spinal deformities in both the coronal and sagittal planes by way of a convex-sided Y shape osteotomy, achieves satisfactory realignment without additional neurological complications, and can be considered an alternative treatment for rigid congenital kyphoscoliosis.

## Background

Congenital kyphoscoliosis is a special type of deformity characterized by a coronal imbalance in the spine and sagittal kyphosis [1]. It is usually a three-dimensional deformity with rotation of the vertebral body and imbalance in the trunk. In addition, abnormal development of the vertebral body and accessory structures often results in poor flexibility. Severe rigid spinal kyphoscoliosis can cause functional impairments, painful costopelvic impingement, and neurologic complications [1, 2]. The surgical treatment of this deformity is challenging for spine surgeons, as it often involves an osteotomy to correct the malalignment in both the sagittal and coronal planes. Traditional pedicle subtraction osteotomy (PSO) can yield approximately 30°–40° of correction in lordosis, which is insufficient to correct this complicated rigid deformity.

Vertebral column resection (VCR) is considered the ultimate surgical technique for these complex deformities, and it involves complete resection of one or more vertebral segments and reconstruction of the anterior column with metal mesh via the combined anterior and posterior approach or the posterior-only approach [3–5]. However, the operation is exhaustively lengthy and has a high risk of major complications and even mortality due to the use of the anterior approach, leading surgeons to investigate alternative methods to VCR [4, 6].

**Fig. 1:**
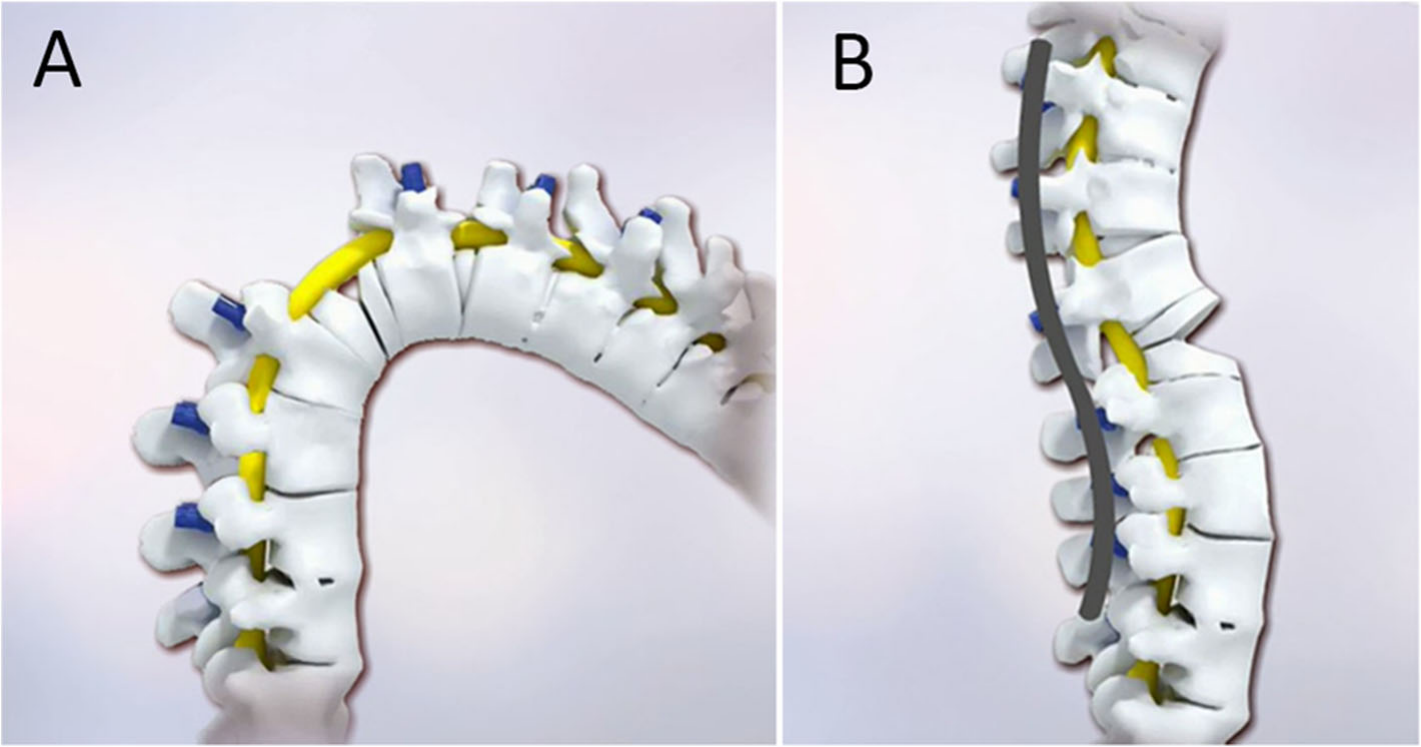
Diagram of Vertebral column decancellation (VCD). **a** Pedicle screws were inserted and ‘Y’ shaped osteotomy was performed. **b** Correction was achieved by elongating the anterior column and shortening the posterior column with the residual bone serving as a “bony cage”

Recently, a new osteotomy technique, vertebral column decancellation (VCD), has been reported to be effective and safe in the correction of sharp angular spinal deformities and ankylosing spondylitis kyphosis [7, 8]. It involves a Y-shaped osteotomy that combines the eggshell technique [9], a Smith-Petersen osteotomy (SPO) [10], a pedicle subtraction osteotomy (PSO) [11] and VCR (Fig. 1). For severe scoliotic deformities, it is necessary to perform asymmetrical osteotomies to achieve correction in the coronal plane [12, 13]. Therefore, the traditional VCD technique was modified to asymmetrical vertebral column decancellation (AVCD) osteotomy which was performed by a large ‘Y’ shape resection on the convex side and a small ‘Y’ shape resection on the concave side. For rigid spinal deformity, however, VCD osteotomy was often performed at multilevel to obtain satisfactory correction. To our knowledge, there was a lack of studies evaluating the results of single level AVCD procedure in patients with rigid congenital kyphoscoliosis.

The purpose of this retrospective cohort study was to evaluate the safety and effectiveness of single level AVCD osteotomy in correcting congenital kyphoscoliosis to determine whether it should be considered a new treatment for these severe rigid curves.

## Methods

After obtaining approval from the ethics committee of our hospital, the charts of patients with rigid congenital kyphoscoliosis treated by AVCD osteotomies at a single level from January 2013 to June 2017 were retrospectively reviewed. The inclusion criteria included an adult age (> 18 years), a diagnosis of congenital kyphoscoliosis with hemivertebra (pure scoliosis cases were excluded), and a minimum follow-up period of 24 months. The exclusion criteria were as follows: 1) idiopathic scoliosis, neuromuscular scoliosis and degenerative scoliosis; 2) abnormal development of the apical vertebrae and complete absence of one or both pedicles. Patients who had previously underwent spine or hip surgery were also excluded. Thirty-one consecutive patients with complete radiographic and follow-up data were included for evaluation. Written informed consent was obtained from all patients. The main complaints of the patients were fatigue or low back pain, cosmetic issues, the inability to stand upright or lie flat, and neurological deficits of the lower extremities.

Full-length, standing anteroposterior and lateral radiographs were taken preoperatively, postoperatively, and at the last follow-up for all patients. Radiographic measurements of the coronal and sagittal deformities were performed on the radiographs using the Cobb method. The correction rates of the Cobb angles in scoliosis and kyphosis were calculated to assess the effectiveness of the operation. In addition, coronal and sagittal balance were measured using the central sacral vertical line and the sagittal vertical axis, as described by Glassman et al. [14]; and the clinical outcome was assessed with the Scoliosis Research Society-22 (SRS-22) questionnaire [15], and visual analogue scale (VAS) score of back pain preoperatively and at the last follow-up. The improvements of them were also documented to evaluate the effectiveness.

To assess the safety of the operation, the operating time, intraoperative blood loss, and complications were recorded. Neurologic deficits were assessed preoperatively, postoperatively, and at the last follow-up by the Asia Spinal Injury Association (ASIA) grading system [16]. All of the data were collected by an independent observer.

### Surgical technique

Osteotomy management was performed in the hemivertebra region in all patients. Both somatosensory-evoked potentials (SEPs) and motor-evoked potentials (MEPs) were continuously monitored during the operation.

Under general anaesthesia, each patient was positioned prone on the operating table. A standard posterior middle incision was made, and the bony structures of the posterior elements were exposed with a subperiosteal dissection. Pedicle screws were then placed in the intended osteotomy site by the freehand technique.

**Fig. 2:**
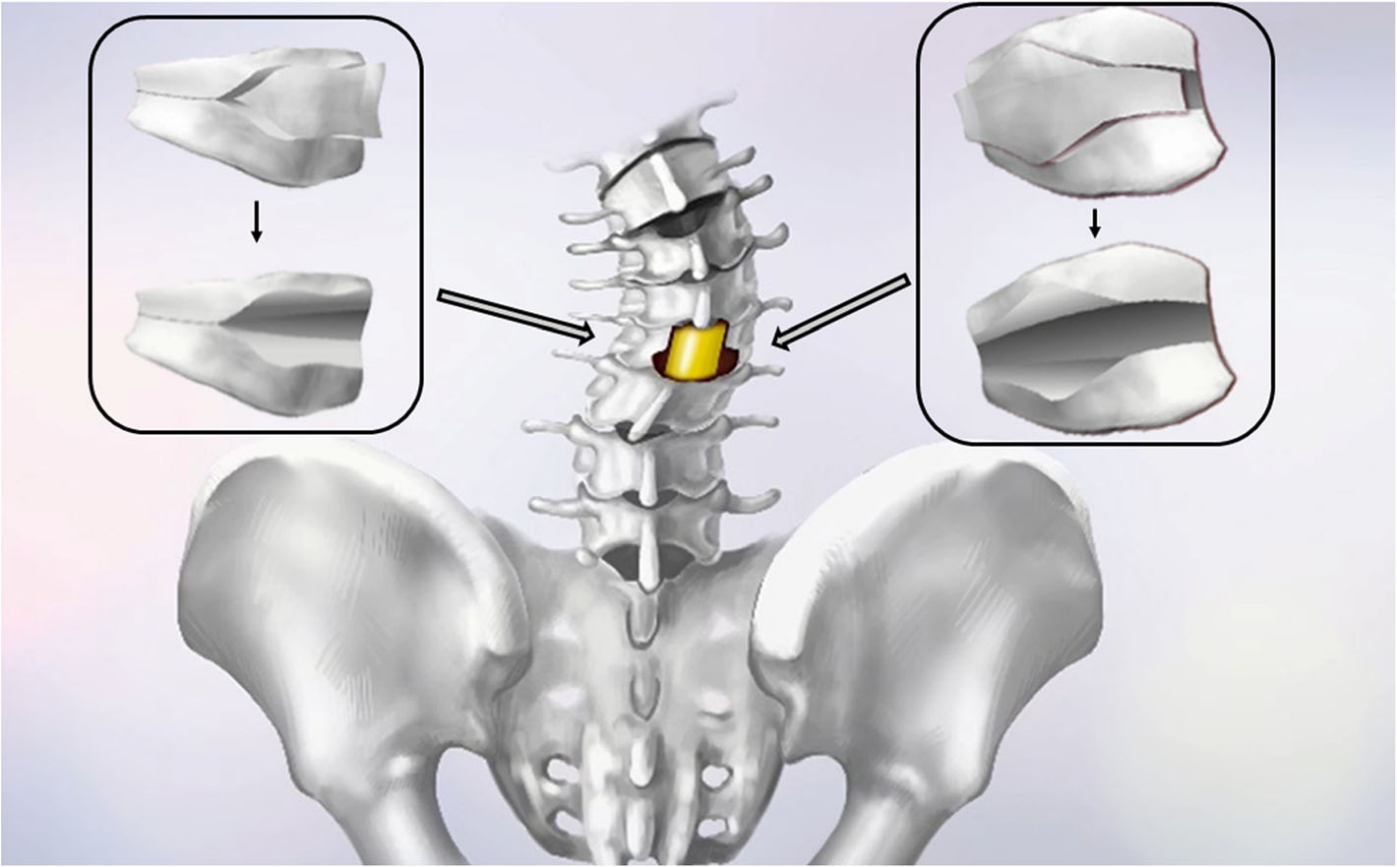
Diagram of asymmetrical operative procedures. Posterior view of the spine with congenital kyphoscoliosis. AVCD osteotomy management was performed in single level of the hemivertebra region. A larger Y shape was resected on the convex side (The box on the right shows the lateral view of the hemivertebra on the convex side), and a smaller Y shape was resected on the concave side (The box on the left shows the lateral view of the hemivertebra on the concave side)

The VCD technique requires that all of the posterior elements (spinous process and lamina) of the corresponding vertebra that are to be osteotomised are removed. The technique was initiated by probing and dilating the pedicles of the deformed vertebrae to gain access to the vertebral body. The pedicle probe and drill were used to enlarge the pedicle holes. A pituitary rongeur or curette was used to remove the cancellous bone of the posterior half of the osteotomy column through the pedicle holes. The posterior and lateral cortical bone sections were adequately removed using a Kerrison rongeur, and a drill was used to thin the anterior cortex. Afterward, the Y shape osteotomy was performed circumferentially, and an appropriate amount of the anterior half and concave side of the cancellous bone was reserved, which served as a correction ‘leverage’ or ‘bony cage’ for the metal mesh in the VCR technique. To perform correction in the coronal plane in kyphoscoliosis patients, a larger Y shape was resected on the convex side with more decancellation than on the concave side for the AVCD technique (Fig. 2). Then, the posterior and convex side of the wedge space of the osteotomy site was closed with manual extension to create an anterior opening wedge and achieve osteoclasis of the anterior cortex and lateral walls. Temporary correction rods were used to avoid unintentional osteotomy closure or significant intraoperative coronal and sagittal translation.

The AVCD technique resulted in appropriate shortening of the posterior and convex side of the column and opening of the anterior column to correct the rigid kyphoscoliosis deformities. The degree of kyphotic correction was adjusted by the location of the hinge in the ‘Y’ shape, while the correction of scoliosis was achieved by reservation of the concave side cortex and resection of the convex side of the cortex of the deformed vertebrae. After correction was confirmed via fluoroscopy, the final internal fixation was completed. A drainage tube was left below the fascia.

Postoperatively, the drainage tube was kept in place until the output fell to < 50 ml/24 h, usually after 3–5 days. The patients were required to use a custom plastic thoracolumbosacral brace during the first 3 months after surgery.

Several measures were taken to reduce bleeding during the operation as follows: 1) An intravenous infusion of tranexamic acid was administered for 20 min before the surgical incision; 2) Intraoperatively, ultrasonic bone cutter was used to reduce bleeding during laminectomy and vertebral resection; 3) Once the spinal canal was opened, fluid gelatine was used in perispinal venous plexus to obtain haemostasis in a timely manner.

### Statistical analysis

All statistical analyses were performed with Statistical Package for Social Sciences software, version 17.0 for Windows (SPSS Inc., Chicago, IL). The data are presented as the means and standard deviations. Paired sample t tests were performed to compare the pre- and postoperative differences. Wilcoxon tests were performed for nonparametric analysis, and the results are expressed as the means and ranges. A *P* value < 0.05 was considered significant.

## Results

There were 15 males and 16 females with a mean age at the time of operation of 36.8 years (range 25–57 years). All patients underwent primary surgeries with the fusion of a mean of 9.1 ± 2.1 levels and a mean follow-up of 29.0 ± 3.5 months. The mean operative time and volume of intraoperative blood loss were 273.9 ± 46.1 min and 782.3 ± 162.6 ml, respectively. The demographic data for the patients included in the study are shown in Table 1.

**Table 1:** Demographic and clinical data

Parameters	Data
Number of patients	31
Gender (M/F)	15/16
Age (years)	36.8 ± 7.6 (25.0–57.0)
Follow-up (months)	29.0 ± 3.5 (24.0–37.0)
blood loss (ml)	782.3 ± 162.6 (500.0–1100.0)
Operation time (minutes)	273.9 ± 46.1 (200.0–370.0)
Fused segment	9.1 ± 2.1 (7.0–15.0)
Osteotomy site	
T10 (n)	2
T11 (n)	3
T12 (n)	4
L1 (n)	12
L2 (n)	8
L3 (n)	2

**Fig. 3:**
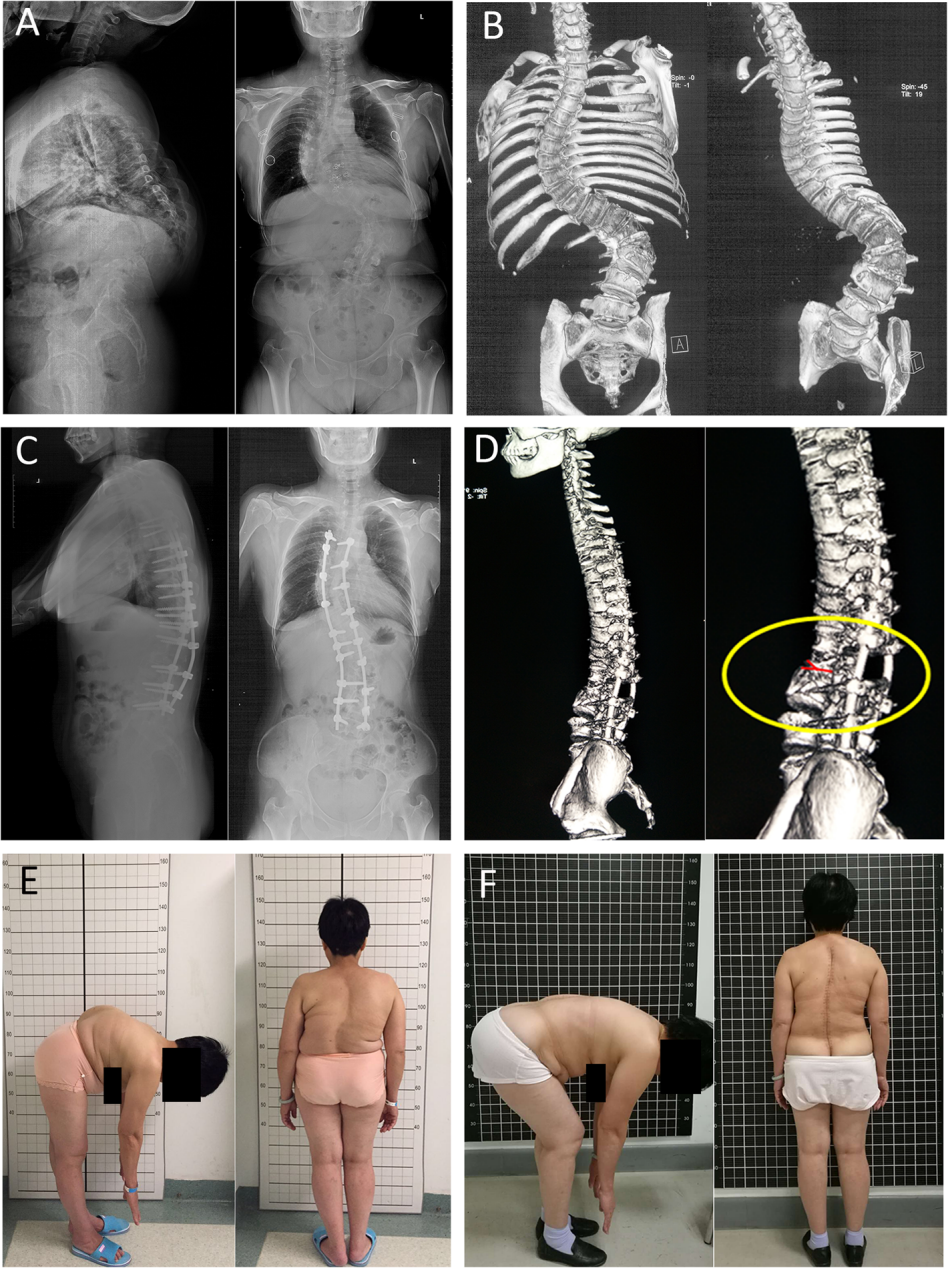
A patient suffering from congenital kyphoscoliosis complained of severe back pain for over 4 years that hardly alleviated with analgesics. **a, b** Preoperative radiograph and CT scan reconstruction showed a remarkable kyphosis of 90° and scoliosis of 104° in thoracolumbar spine. **c** Asymmetrical vertebral column decancellation (AVCD) was performed at L2. The sagittal and coronal profile was improved to 25° and 35°as shown in the two-year follow-up images. **d** The circle in the lower right panel shows the Y-shaped osteotomy. **e, f** Pre- and Post-operative lateral view shows that the cosmetic disfigurement was improved obviously

The average degree of scoliosis (main curve) was measured to be 81.4° preoperatively (range, 53°–110°) and 24.7° (range, 10°–53°) postoperatively, with a correction rate of 69.7%. The average degree of local kyphosis was 86.5° (range, 68°–107°) preoperatively and 29.2° (range, 12°–48°) postoperatively, with a correction rate of 66.2%. The mean coronal balance was 28.9 mm (range, 10.2–54.2 mm) preoperatively and 7.6 mm (range, 2.0–20.6 mm) postoperatively, showing improvement. The sagittal balance improved from 72.3 mm (range, 33.8–97.2 mm) before surgery to 16.9 mm (range, 5.6 to 31.1 mm) postoperatively. All patients exhibited solid fusion at the final follow-up, according to the radiological evidence, without obvious loss of correction (Table 2) (Fig. 3).

**Table 2:** Summary of clinical and radiologic outcomes

*n* = 31	Preoperative	Final follow-up	*P*
Coronal main curve (°)	81.4 ± 14.0 (53.0–110.0)	24.7 ± 8.8 (10.0–53.0)	< 0.001
coronal balance (mm)	28.9 ± 11.9 (10.2–54.2)	7.6 ± 4.7 (2.0–20.6)	< 0.001
local kyphosis (°)	86.5 ± 9.8 (68.0–107.0)	29.2 ± 7.7 (12.0–48.0)	< 0.001
Sagittal balance (mm)	72.3 ± 17.6 (33.8–97.2)	16.9 ± 7.5 (5.6–31.1)	< 0.001
VAS score	2.6 ± 2.2 (0.0–6.0)	0.8 ± 1.0 (0.0–3.0)	< 0.001
ASIA scale			
A(n)	0	0	
B(n)	0	0	
C(n)	2	0	
D(n)	10	4	
E(n)	19	27	

*VAS* visual analog scale, *SRS*-22 Scoliosis Research Society-22 questionnaire, *ASIA* Asia Spinal Injury Association

The average preoperative VAS score for back pain was 2.6, and it reduced to 0.8 at the 2-year follow-up. There were also statistically significant improvements from before the operation to the final evaluation in the scores for all domains of the SRS-22 questionnaire (Tables 2 and 3) .

**Table 3:** SRS-22 outcomes data

SRS-22	Preoperative	Final follow-up	*P*
Function	3.3 ± 0.6 (2.9–3.8)	4.4 ± 0.5 (4.0–5.0)	< 0.001
Pain	2.6 ± 0.7 (2.0–3.2)	3.6 ± 0.6 (3.2–5.0)	< 0.001
Appearance	2.7 ± 0.5 (2.2–3.4)	3.8 ± 0.6 (3.1–5.0)	< 0.001
Mental	3.2 ± 0.5 (2.6–3.8)	4.0 ± 0.4 (3.5–5.0)	< 0.001
Satisfaction	–	4.2 ± 0.6 (3.0–5.0)	–

*SRS-22* Scoliosis Research Society-22 questionnaire

Complications were encountered in 5 patients, including 2 cases of wound problems and 3 cases of cerebrospinal fluid (CSF) leakage due to dural tears. The cases of CSF leakage in resolved without additional sequelae after lumbar puncture and continuous lumbar cisterna drainage for 8–10 days. One patient, whose preoperative ASIA scale was grade C, suffered transient paralysis in the right lower extremity, which resolved within 4 weeks. No permanent postoperative neurologic complications occurred. No major acute complications, such as death, occurred in this patient group. No delayed complications, such as pseudarthrosis or screw misplacement, were detected during the follow-up period.

## Discussion

Adult congenital kyphoscoliosis is considered a persistent and perplexing problem because most of the deformities are rigid by nature [17, 18]. This three-dimensional deformity involves both the coronal and sagittal planes. In contrast to adolescent scoliosis, which is usually asymptomatic, back pain is a common clinical presentation in adult scoliosis patients [19, 20]. In addition, adult patients are more likely to experience neurological dysfunction from canal compromise and coronal or sagittal malalignment. It has been challenging for spine surgeons to treat adult severe rigid congenital kyphoscoliosis. To obtain a balanced spine, a major spinal osteotomy needs to be performed for most of these patients [21, 22].

VCR is considered the ultimate surgical technique for spinal deformity correction and is generally utilized for cases with severe, rigid spinal scoliosis and kyphosis. This procedure was called a “formidable last resort technique for the most tenacious spinal deformities” by Suk et al. [23] VCR initially involved a posterior-only approach. Afterward, simultaneous or staged anterior-posterior approaches were introduced and gradually became the gold standard in the treatment of rigid deformities of the spine [3, 4, 24]. However, this powerful method can be technically difficult to perform and have a high risk for complications, particularly in the correction of kyphotic deformities. Regardless of whether it is performed in an open or endoscopic manner, the anterior approach is associated with increased pulmonary complications, increased intraoperative bleeding and a prolonged surgical time. Although VCR has been considered the most powerful method for the correction of deformities, the disadvantages have led surgeons to investigate alternative methods to VCR. As an alternative to VCR, multilevel VCD for the treatment of congenital spinal deformity was first reported by Wang et al. [7] in 2011. However, with the continuous standardization of the Y-shaped osteotomy, single level VCD can now achieve comparable correction to VCR. To our knowledge, the safety and effectiveness of single level AVCD procedure has not been reported in patients with rigid congenital kyphoscoliosis.

In this study, 31 adult patients with severe congenital kyphoscoliosis underwent AVCD at single level. Significant corrections in the major Cobb angles were obtained in both the scoliosis (69.7%) and kyphosis (66.2%) curves, which is equivalent to the results of VCR that were previously reported. Suk et al. [23] reported that the improvements in deformity correction were 67% in scoliotic curves and 42% in kyphotic curves in a series of 38 adult patients treated by VCR. Riley et al. [25] reported an improvement in the major Cobb angle, with 53.9% correction for the adult cohort treated by PVCR. Therefore, AVCD at single level can be as effective as traditional VCR surgery. In addition, significant improvements in the SRS-22 domain scores were observed. Our final follow-up data showed an average SRS-22 score of 4.0, which was similar to that of patients who underwent simpler spine surgeries. The total SRS-22 score postoperatively was observed to be 4.27 in a study in adolescent idiopathic scoliosis patients [26]. Overall, 29 of all the 31 patients (93.5%) were satisfied with their surgical management and reported that they would choose to repeat the procedure if necessary at a minimum follow-up of 2 years.

In terms of surgical safety, the mean volume of intraoperative blood loss was 782.3 ± 162.6 ml, which was well below those previously reported for VCR, with an average blood loss of > 2 L being reported in many series studies [23, 27, 28]. The mean operative time was 273.9 ± 46.1, which may also increase the safety of the operation compared with VCR, which was reported to have an operative time of 420.6 ± 90.7 min in an adult cohort in a study by Riley et al. [25]. Lenke et al. [29] reported 40% overall and 11.4% neurologic-only complication rates, and Suk et al. [23] reported a 29% complication rate for VCR. However, of the 31 patients in this study, only 5 (16.1%) sustained complications, including wound problems and CSF leakage. No permanent postoperative neurologic complications occurred. Most importantly, no pseudarthroses or neurological damage were found in any of the 31 patients by the follow-up, which was attributed to the middle column being retained, a small amount of spine cord shortening and obviation of mesh reconstruction.

As the Y-shaped osteotomy technique is performed for rigid sagittal plane deformities, such as ankylosing spondylitis (AS) kyphosis and Pott’s kyphosis, and VCD is characterized by controlled anterior column opening, posterior column closing and middle column preservation for the hinge [7, 8, 30]. Compared with the traditional V shape osteotomy, this Y shape technique removes a relatively smaller amount of the posterior half of the osteotomy column and preserves more of the middle column as the hinge, which serves as a “bony cage” to provide greater stability instantly and better fusion in the future. Most of the patients with severe rigid kyphosis, such as Pott’s deformity and AS kyphosis, have ideal indications for the Y-shaped VCD osteotomy. The goals of the AVCD technique are to preserve as much as possible of the bone on the concave side and middle column, which may take the place of the metal mesh described in the VCR technique. The residual bone of the deformed vertebrae serves as correction “leverage” to facilitate deformity correction. Opening of the anterior column achieves a rational correction angle and decreases the need for shortening of the posterior column, which reduces the risk of neurological sequelae. In addition, it is not necessary to expose the segmental vessels in most AVCD procedures, which is different from VCR and reduces the risk of vascular complications and intraoperative blood loss.

Compared with previous multilevel VCD, the hemivertebra region was selected for single level AVCD osteotomy in this study, which achieved satisfactory correction in patients with rigid congenital kyphoscoliosis. In addition, because of fewer osteotomy segments and more vertebral bone retained by the Y-shaped osteotomy, the volume of intraoperative blood loss was significantly lower than other previously reported surgical methods. And no patients suffered neurological complication by the follow-up in this study, which proves the safety of the AVCD procedure. These advantages of AVCD may make it more widely used in the future.

The AVCD osteotomy is suitable for patients with severe rigid congenital deformities that cannot be treated with less invasive procedures, such as Smith-Peterson or wedge osteotomy. However, not all complex congenital kyphoscoliosis deformities can be treated with AVCD. In our opinion, at least two relative contraindications of AVCD should be noted: 1) severe rotation of the apical vertebra, with the concave pedicles rotated towards the ventral side of the spine, which makes it impossible to fix the adjacent pedicles and expose the periapical pedicles; and 2) pedicle dysplasia involving multiple segments of the spine, making screw insertion and spinal stability preservation impossible. Furthermore, for specific patients with severe rigid deformities, two-level AVCD needs to be performed for satisfactory correction.

There are several limitations in this study. One of the limitations is that it is a single institutional retrospective study. Limitations are also lied in the relatively small sample size and short follow-up periods. Future prospective studies (such as RCTs) are needed to fully demonstrate the outcome of AVCD osteotomy in patients with severe rigid congenital deformities.

## Conclusions

AVCD is a safe and reliable procedure that overcomes the problems associated with treating rigid kyphoscoliosis. Single level AVCD yields satisfactory realignment of deformed spines, successful fusion, an acceptable volume of intraoperative blood loss, and favourable clinical outcomes without additional neurological complications. However, AVCD is a technically demanding procedure that should be performed by the most experienced spine surgeons.

## Supplementary information

**Additional file 1: Table 1.** Demographic data. **Table 2**. Radiographic data.

## Data Availability

The datasets analyzed during the current study are available from the corresponding author on reasonable request.

## Abbreviations

PSO: Pedicle subtraction osteotomy
VCR: Vertebral column resection
VCD: Vertebral column decancellation
AVCD: Asymmetrical vertebral column decancellation
SPO: Smith-Petersen osteotomy
SRS-22: Scoliosis Research Society-22
VAS: Visual analogue score
ASIA: Asia Spinal Injury Association
SEPs: Somatosensory-evoked potentials
MEPs: Motor-evoked potentials
CSF: Cerebrospinal fluid
AS: Ankylosing spondylitis
