# Supplementary material for: Asymmetrical vertebral column decancellation for the management of rigid congenital kyphoscoliosis
Source: BMC Musculoskelet Disord. 2020 Aug 17;21:555. doi: 10.1186/s12891-020-03558-x (PMC7433174; doi:10.1186/s12891-020-03558-x)
Supplement: Supplementary file 1 — Additional file 1: Table 1. Demographic data. Table 2. Radiographic data. [file 12891_2020_3558_MOESM1_ESM.docx]

Table 1 Demographic data

| Case | Gender | Age (y) | Follow-up (mon) | Hemi site | Osteotomy site | blood loss (ml) | Operation time (min) | Fused segment |
| --- | --- | --- | --- | --- | --- | --- | --- | --- |
| 1 | F | 26 | 31 | T11 | T11 | 500 | 200 | 7 |
| 2 | F | 43 | 28 | L2 | L2 | 700 | 270 | 11 |
| 3 | M | 38 | 29 | L1 | L1 | 650 | 210 | 8 |
| 4 | F | 29 | 35 | T12/L1 | L1 | 800 | 300 | 9 |
| 5 | M | 41 | 30 | L1 | L1 | 1100 | 310 | 12 |
| 6 | M | 31 | 25 | L2 | L2 | 600 | 255 | 8 |
| 7 | F | 40 | 24 | L1 | L1 | 850 | 280 | 10 |
| 8 | F | 52 | 27 | T11 | T11 | 900 | 300 | 8 |
| 9 | M | 30 | 26 | L3 | L3 | 550 | 220 | 9 |
| 10 | F | 37 | 31 | L1 | L1 | 1100 | 315 | 15 |
| 11 | M | 29 | 28 | T12 | T12 | 950 | 240 | 11 |
| 12 | M | 33 | 33 | L1/L2 | L1 | 800 | 270 | 9 |
| 13 | M | 45 | 30 | L1 | L1 | 700 | 265 | 9 |
| 14 | F | 57 | 28 | L1/L2 | L2 | 850 | 240 | 14 |
| 15 | F | 39 | 35 | L2 | L2 | 750 | 290 | 8 |
| 16 | M | 29 | 27 | T12 | T12 | 900 | 245 | 7 |
| 17 | F | 45 | 29 | T10 | T10 | 1000 | 305 | 13 |
| 18 | F | 38 | 37 | L1 | L1 | 650 | 230 | 8 |
| 19 | F | 40 | 28 | T11/T12 | T12 | 800 | 210 | 9 |
| 20 | M | 36 | 26 | L2 | L2 | 500 | 200 | 8 |
| 21 | F | 44 | 30 | L1 | L1 | 750 | 285 | 8 |
| 22 | M | 42 | 24 | L1 | L1 | 550 | 250 | 7 |
| 23 | M | 37 | 33 | L1/L2 | L2 | 750 | 365 | 8 |
| 24 | F | 40 | 35 | L1 | L1 | 850 | 355 | 7 |
| 25 | M | 29 | 26 | L2/L3 | L3 | 650 | 310 | 8 |
| 26 | M | 25 | 24 | T9/T10 | T10 | 700 | 290 | 7 |
| 27 | F | 31 | 31 | T11 | T11 | 900 | 280 | 7 |
| 28 | F | 34 | 25 | L2 | L2 | 700 | 370 | 8 |
| 29 | F | 43 | 28 | T12 | T12 | 1000 | 325 | 11 |
| 30 | M | 30 | 30 | L2 | L2 | 950 | 270 | 9 |
| 31 | M | 28 | 26 | L1 | L1 | 800 | 235 | 10 |

Table 2 Radiographic data

| Case | Coronal main curve | | coronal balance | | local kyphosis | | Sagittal balance | | VAS | |
| --- | --- | --- | --- | --- | --- | --- | --- | --- | --- | --- |
|  | Preop (°) | 2y-Postop (°) | Preop (mm) | 2y-Postop (mm) | Preop (°) | 2y-Postop (°) | Preop (mm) | 2y-Postop (mm) | Preop | 2y-Postop |
| 1 | 97 | 34 | 30.6 | 8.1 | 85 | 19 | 93.2 | 19.4 | 0 | 0 |
| 2 | 85 | 27 | 21.4 | 10.2 | 92 | 31 | 81.7 | 30.3 | 6 | 2 |
| 3 | 88 | 16 | 17.0 | 4.3 | 88 | 17 | 75.8 | 21.2 | 3 | 1 |
| 4 | 110 | 53 | 54.2 | 20.6 | 75 | 27 | 59.3 | 19.0 | 0 | 0 |
| 5 | 95 | 25 | 28.1 | 7.0 | 84 | 30 | 39.6 | 8.7 | 5 | 2 |
| 6 | 74 | 19 | 36.5 | 15.7 | 91 | 33 | 78.9 | 13.5 | 1 | 0 |
| 7 | 82 | 20 | 19.5 | 3.3 | 70 | 28 | 62.5 | 9.1 | 0 | 0 |
| 8 | 70 | 23 | 21.7 | 10.8 | 68 | 22 | 86.1 | 10.1 | 4 | 0 |
| 9 | 78 | 17 | 16.9 | 6.4 | 79 | 16 | 68.4 | 7.8 | 0 | 0 |
| 10 | 83 | 27 | 10.2 | 2.1 | 81 | 39 | 50.9 | 10.4 | 6 | 1 |
| 11 | 87 | 29 | 42.3 | 14.5 | 94 | 35 | 88.3 | 17.3 | 4 | 1 |
| 12 | 92 | 26 | 39.1 | 8.9 | 107 | 32 | 50.1 | 21.6 | 0 | 0 |
| 13 | 77 | 29 | 33.0 | 9.0 | 73 | 29 | 97.2 | 24.1 | 3 | 0 |
| 14 | 104 | 35 | 38.2 | 6.5 | 90 | 25 | 45.6 | 10.2 | 5 | 2 |
| 15 | 85 | 33 | 27.3 | 3.7 | 78 | 12 | 81.1 | 14.8 | 2 | 1 |
| 16 | 96 | 21 | 41.7 | 7.2 | 89 | 29 | 72.7 | 9.4 | 0 | 0 |
| 17 | 71 | 20 | 25.2 | 5.5 | 95 | 34 | 67.5 | 16.5 | 2 | 0 |
| 18 | 67 | 15 | 14.8 | 2.0 | 82 | 30 | 33.8 | 5.6 | 3 | 1 |
| 19 | 80 | 29 | 46.8 | 12.7 | 103 | 40 | 54.7 | 12.9 | 0 | 0 |
| 20 | 58 | 17 | 11.4 | 3.5 | 86 | 34 | 96.4 | 31.1 | 4 | 3 |
| 21 | 71 | 24 | 23.3 | 4.6 | 90 | 23 | 78.7 | 17.0 | 0 | 0 |
| 22 | 63 | 18 | 15.0 | 2.3 | 97 | 31 | 95.8 | 25.8 | 6 | 1 |
| 23 | 97 | 32 | 40.5 | 8.1 | 78 | 32 | 73.4 | 8.5 | 5 | 3 |
| 24 | 102 | 40 | 43.3 | 10.5 | 87 | 35 | 93.8 | 24.7 | 6 | 2 |
| 25 | 93 | 30 | 35.7 | 5.0 | 92 | 28 | 81.7 | 20.6 | 3 | 0 |
| 26 | 78 | 25 | 36.5 | 3.2 | 72 | 26 | 69.4 | 11.0 | 0 | 0 |
| 27 | 62 | 11 | 20.6 | 9.3 | 96 | 24 | 90.8 | 23.1 | 5 | 1 |
| 28 | 53 | 10 | 13.8 | 2.9 | 103 | 48 | 79.7 | 28.5 | 0 | 0 |
| 29 | 82 | 23 | 43.6 | 17.8 | 93 | 42 | 68.5 | 13.0 | 4 | 2 |
| 30 | 74 | 22 | 29.0 | 7.5 | 80 | 25 | 77.8 | 29.1 | 3 | 0 |
| 31 | 69 | 17 | 17.3 | 3.8 | 85 | 28 | 47.6 | 9.3 | 2 | 1 |
